# Dependence of Modified Butterworth Van-Dyke Model Parameters and Magnetoimpedance on DC Magnetic Field for Magnetoelectric Composites

**DOI:** 10.3390/ma14164730

**Published:** 2021-08-21

**Authors:** Lei Chen, Yao Wang

**Affiliations:** 1 Key Lab of Computer Vision and Intelligent Information System, Chongqing University of Arts and Sciences, Chongqing 402160, China; 20060012@cqwu.edu.cn; 2 School of Electronic Information and Electrical Engineering, Shanghai Jiao Tong University, Shanghai 200240, China

**Keywords:** magnetostriction, modified Butterworth-Van Dyke model, magnetoimpedance effect, mechanical quality factor, magnetoelectric composite

## Abstract

This study investigates the impedance curve of magnetoelectric (ME) composites (i.e., Fe_80_Si_9_B_11_/Pb(Zr_0.3_Ti_0.7_)O_3_ laminate) and extracts the modified Butterworth–Van Dyke (MBVD) model’s parameters at various direct current (DC) bias magnetic fields *H*_dc_. It is interesting to find that both the magnetoimpedance and MBVD model’s parameters of ME composite depend on *H*_dc_, which is primarily attributed to the dependence of FeSiB’s and neighboring PZT’s material properties on *H*_dc_. On one hand, the delta E effect and magnetostriction of FeSiB result in the change in PZT’s dielectric permittivity, leading to the variation in impedance with *H*_dc_. On the other hand, the magnetostriction and mechanical energy dissipation of FeSiB as a function of *H*_dc_ result in the field dependences of the MBVD model’s parameters and mechanical quality factor. Furthermore, the influences of piezoelectric and electrode materials properties on the MBVD model’s parameters are analyzed. This study plays a guiding role for ME sensor design and its application.

## 1. Introduction

Magnetoelectric (ME) materials produce strong ME effects due to the mechanical coupling between magnetostrictive and piezoelectric materials, which has been studied intensively in both theories and experiments [1,2,3,4,5,6]. Such ME effects provide a promising candidate for the highly sensitive DC magnetic field sensor due to its significant variations with external direct current (DC) magnetic field. Dong et al. [7] presented a ME laminate under a constant drive of *H*_ac_ = 1 Oe, which can reach the limit of detection (LOD) for a DC magnetic field *H*_dc_ of 10^−4^ Oe. Sun et al. [2] reported a novel Nano-Electromechanical System (NEMS) AlN/FeGaB resonator with a high DC magnetic field sensitivity of 280 kHz/Oe and a LOD of 8 × 10^−6^ Oe. Liu et al. [8] demonstrated a highly sensitive DC magnetic field sensor with a LOD of 2 × 10^−5^ Oe. Martins et al. [9] showed a Metglas/poly(vinylidene fluoride)/Metglas magnetoelectric laminate with the sensitivity of 30 mV·Oe^−1^ and resolution of 8 μ Oe for *H*_dc_ detection, and its correlation coefficient, linearity and accuracy values reached 0.995, 95.9% and 99.4%, respectively. Yao et al. [10] developed a Metglas/PMNT/Metglas laminate with the LOD of 10 × 10^−5^ Oe for *H*_dc_ detection. Wang et al. [11] also proposed a transformer-type magnetic sensor consisting of soft magnetostrictive alloy FeBSiC/piezoelectric ceramics Pb(Zr,Ti)O_3_/FeBSiC heterostructure wrapped with both the exciting and sensing coils, which provided the maximum magnetic field sensitivity of 2.12 V/Oe and equivalent magnetic noise of 114 × 10^−8^ Oe/$\sqrt{\mathrm{HZ}}$ (at 1 Hz).

Meanwhile the material property and structure of ME composite have been researched intensively for the magnetic sensor application. As such, the field-dependent characteristics of piezomagnetic coefficient for magnetostrictive material [12], the effect of different magnetostrictive materials on the *H*_dc_ sensitivity [13,14,15,16], and the optimum structure of piezoelectric/magnetostrictive composite [17,18,19,20] etc. were reported. Additional to understanding the material property of ME composite, it is essential to study the equivalent electrical parameters of ME composite to further improve the DC magnetic sensor performance. However, few articles have reported and analyzed the *H*_dc_ dependence of equivalent electrical parameters based on the modified Butterworth–Van Dyke (MBVD) model of magnetoelectric material, even though this is crucial to guide the conditioning circuit design of the ME sensor. Hence, the exploration of electrical equivalent circuit for ME device in this study facilitates understandings of corresponding electrical resonance behavior, which is beneficial for the design and optimization of impedance matching circuits for ME devices. Additionally, this study is expected to guide the design of the magnetic-field-tuned ultrasonic transducer, which can effectively solve the problem of resonance frequency shift and impedance mismatch of the ultrasonic transducer. It is noted that the MBVD model characterizes the loss mechanisms more accurately compared to the conventional Butterworth–Van Dyke model by considering the effects of additional electrical losses and dielectric losses, which can model the measured results more accurately.

In this paper, we investigate the equivalent circuit of the ME sensor based on the MBVD model of PZT/FeSiB laminated composite. It is noted that Lead zirconate titanate Pb(Zr_0.3_Ti_0.7_)O_3_ (PZT) exhibits the outstanding piezoelectric performance and high mechanical quality factor compared to other piezoelectric materials such as polyvinylidene fluoride (PVDF) and BaTiO_3_ etc. Meanwhile, the FeSiB (International standard trademark Metglas-2605 S2) possesses a low saturation field and a strong magnetostrictive effect at low magnetic biases *H*_dc_ due to its ultrahigh magnetic permeability (i.e., the initial magnetic permeability of 45,000). Correspondingly, magnetostrictive material FeSiB and piezoelectric material PZT are utilized for the ME composite in order to obtain highly magnetic sensing capabilities. In this study, the electrical equivalent circuit parameters of the ME sensor are calculated with the electrical resonance characteristics of measured impedance. Furthermore, the dependences of magnetoimpedance and corresponding MBVD model’s parameters on DC magnetic field are measured and discussed. Such dependences are mainly attributed to the delta E and magnetostrictive effects of FeSiB and correspondingly varied PZT’s dielectric permittivity. Additionally, the effects of the piezoelectric materials and electrode material’s properties on the MBVD model’s parameters are analyzed. The study of electrical equivalent circuit facilitates the understanding of electrical resonance behavior for ME devices, and it plays a crucial role in the design of impedance matching circuits for ME devices. Meanwhile, the controllable impedance and dielectric permittivity of PZT/FeSiB ME composites with DC bias magnetic field have broad potential applications, such as tunable spin filters, storage devices, and magnetic sensor etc.

## 2. Experiment

The ME sensor consists of PZT/FeSiB laminated composite, where the sizes of magnetostrictive (FeSiB, supplied by Foshan Huaxin Microlite Metal Co., Ltd., Foshan, China) layer and the piezoelectric (PZT, produced by Zibo Yuhai Ceracomp Co., Ltd., Zibo, China) layer are 12 mm × 5 mm × 0.03 mm and 12 mm × 6 mm × 0.8 mm, respectively. First, the PZT plate and FeSiB ribbon are dipped in organic impregnant to clean them. Subsequently, the soft magnetic ribbon FeSiB is bonded with PZT plate by using epoxy glue. Here the West System 105/206 resin/hardener epoxy with a good mechanical property and a low viscosity is utilized to provide strong bondings among layers. The mixture ratio for the epoxy part ‘Resin’ and part ‘hardener’ is specified as 5:1 by the supplier. Then the PZT/FeSiB laminated composite is compacted in a vacuum bag and cured for 12 h at room temperature to further guarantee the strong bonding among layers. The thickness of the epoxy layers is controlled to be less than 5 μm with vacuum bagging techniques, which has been proved to negligibly affect the ME performance, according to previous research [19]. Considering the ease of fabrication and ME performance, the PZT/FeSiB laminated composite is designed to operate in the L–T (i.e., longitudinal–transverse) mode. That is to say, the FeSiB layer is magnetized along the longitudinal direction (i.e., length direction) since the demagnetizing field is much smaller along this direction. Meanwhile the silver electrodes of piezoelectric layer are at its top and bottom surfaces, and the PZT is poled along the transverse direction (i.e., thickness direction).

To measure the impedance of ME composite as a function of the external DC magnetic field *H*_dc_, *H*_dc_ is applied along the longitudinal direction of FeSiB layer with a pair of electromagnets driven by a SR830 Lock-In Amplifier. Here the *H*_dc_ varies from 0 to 400 Oe, which is calibrated with a Gauss magnetometer (Lake Shore 455 DSP, Columbus, OH, USA). Additionally, when analyzing the dielectric characteristics of the ME sensor, an Impedance Analyzer (4194 A HP Agilent, Santa Clara, CA, USA) is used to measure the magnetoimpedance (Z) of ME composite with the excitation frequency ranged from 125 kHz to 155 kHz.

## 3. Results and Discussion

**Figure 1:**
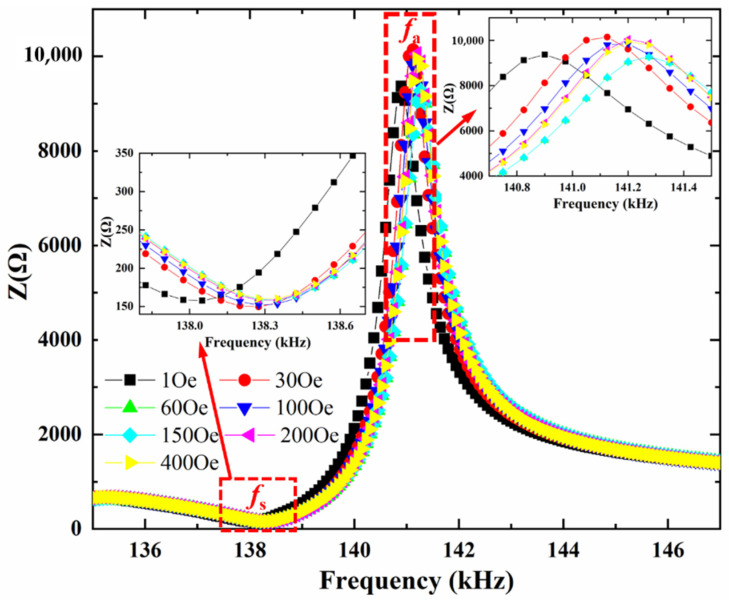
Impedance curve of the ME sensor at various bias DC magnetic fields, and the insets show enlarged details around the maximum and minimum impedances. The maximum relative standard deviations (RSD, i.e., standard deviation/mean × 100%) of impedance with multiple measurements is 0.27%.

Figure 1 shows the impedance Z of the ME sensor as a function of electrical excitation frequency *f* when the varied DC bias magnetic field is applied along the length direction. As illustrated in the inset of Figure 1, the maximum and minimum impedance as a function of excitation frequency show a strong dependence on DC bias magnetic field.

It is known that the impedance of the ME sensor is defined by [21],

(1) $$Z=\sqrt{\frac{\mu_{0}\mu_{eff}}{\varepsilon_{0}\varepsilon_{eff}}}$$

where $\mu_{eff}$ and $\varepsilon_{eff}$ are the effective relative permeability and permittivity, $\mu_{0}$ and $\varepsilon_{0}$ are vacuum permeability and permittivity, respectively. The effective relative permittivity $\varepsilon_{eff}$ can be represented as [22].

(2) $$\varepsilon_{eff}=\varepsilon_{r}+d_{31,p}^{2}E_{m}\left[\frac{n_{pzt}E_{pzt}}{\left(1-n_{pzt}\right)E_{m}+n_{pzt}E_{pzt}}\frac{\tan\left(\frac{\pi f}{2f_{s}}\right)}{\frac{\pi f}{2f_{s}}}-1\right]$$

where $\varepsilon_{r}$ is relative permittivity of piezoelectric material, *d*_31,*p*_ is the piezoelectric coefficient, *f_s_* is the resonance frequency, $E_{pzt}$ and $E_{m}$ are the Young’s modulus of piezoelectric and magnetostrictive materials, respectively. $n_{pzt}$ and $1-n_{pzt}$ are the volume fractions of piezoelectric material PZT and magnetostrictive material FeSiB in the ME sensor, respectively.

By applying a DC bias magnetic field to the magnetostrictive material FeSiB, the magnetostriction is produced by FeSiB and transferred to the PZT layer through interfacial coupling. Meanwhile the magnetostrictive stress will also change the Young’s modulus $E_{m\;}$ of magnetostrictive material FeSiB and corresponding resonance frequency $f_{s}$. Correspondingly from the inset of Figure 1, the electromechanical resonance frequency *f_s_* of the ME sensor shows a strong dependence on DC bias magnetic field *H*_dc_. Specifically, the resonance frequency *f_s_* of the ME sensor is determined by the geometrical dimensions and material parameters (i.e., Young’s modulus and mass density) of both piezomagnetic and piezoelectric materials, and is expressed as [12],

(3) $$f_{s}=\frac{1}{2l}\sqrt{\frac{\bar{E}}{\bar{\rho }}}$$

where *l* is the length of the ME sensor, $\bar{\rho }$ and $\bar{E}$ are the average density and equivalent Young’s modulus of ME laminate, respectively. For the ME composite, $\bar{E}$ and $\bar{\rho }$ are determined by [6],

(4) $$\bar{E}=\left(1-n_{pzt}\right)E_{m}+n_{pzt}E_{pzt}$$

(5) $$\bar{\rho }=\left(1-n_{pzt}\right)\rho_{m}+n_{pzt}\rho_{pzt}$$

where $\rho_{pzt}$ and $\rho_{m}$ are the densities of piezoelectric and magnetostrictive materials, respectively.

Here the Young’s modulus of magnetostrictive material FeSiB is given by [23],

(6) $$E_{m}=\frac{\sigma }{s^{e}+s^{me}}$$

where $s^{e}$, σ,$\;s^{me}$ are the elastic strain, elastic stress and magnetoelastic strain, respectively. The magnetoelastic strain arises from the magnetic domain reorientation during the varied *H*_dc_ [24,25], which results in the change in effective Young’s modulus with *H*_dc_. As a result, the shifts in corresponding resonance frequency (Equation (3)) with *H*_dc_ are observed.

According to Equations (1) and (2), the variations in the Young’s modulus $E_{m\;}$ of FeSiB and resonance frequency $f_{s}$ with *H*_dc_ also lead to the changes in effective relative permittivity and corresponding impedance with *H*_dc_ for ME composite. It is noted that the combination of magnetoresistance (MR) and the Maxwell–Wagner effect could also cause the magnetodielectric effect, according to the previous report [26]. However, for our asymmetric PZT/FeSiB laminate, piezoelectric material PZT is covered with the insulating epoxy glue at surface to prevent the current penetrating into the neighboring magnetic ribbon FeSiB. Hence, there is no giant magnetoresistance effect since the sensing current cannot go through the magnetic layers and, correspondingly, no spin dependent scattering phenomenon happens in the ferromagnetic layer. Furthermore, Castel et al. [22] have also reported that the magnetodielectric effect of BaTiO_3_-Ni laminated composite could reach 10% near the resonance frequency at *H*_dc_ = 6 kOe and clarified that the magnetodielectric mechanism of their composites was based on the strain effect instead of the Maxwell—Wagner effect.

**Figure 2:**
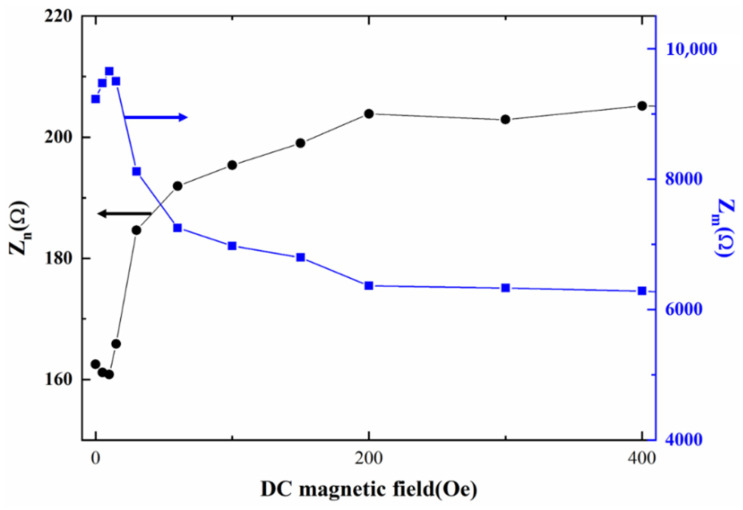
The maximum impedance Z_m_ and minimum impedance Z_n_ as a function of DC magnetic field. The maximum RSDs of maximum impedance and minimum impedance with multiple measurements are 0.24% and 0.25%, respectively.

It is also interesting to find in Figure 2 that the maximum impedance Z_m_ at the antiresonance frequency (*f_a_*) increases to a maximum value at *H*_dc_ = 30 Oe, and then decreases with further increasing *H*_dc_, while the minimum impedance Z_n_ at the resonance frequency (*f_s_*) varies in the opposite trend. Namely, Z_n_ decreases to a minimum value, and then increases with the increasing *H*_dc_. This is mainly because the capacitance is directly proportional to dielectric permittivity, the minimum capacitance value at *f_a_* results in the maximum impedance Z_m_ and the maximum capacitance value at *f_s_* leads to the minimum impedance Z_n_ according to Equation (1).

**Figure 3:**
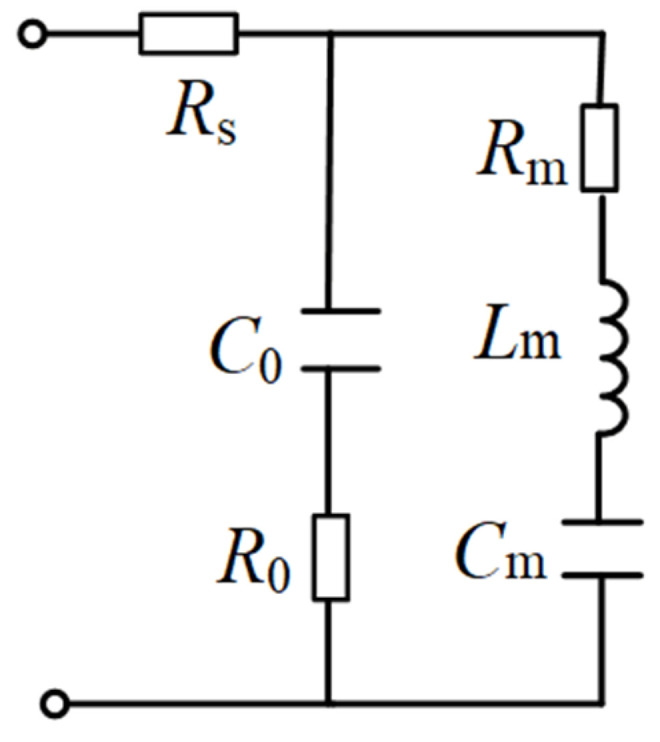
The modified Butterworth–Van Dyke (MBVD) circuit for the ME sensor.

In order to understand the trend of impedance as a function of *DC* magnetic field, the electromechanical (ME) sensor is characterized with a lumped-parameter equivalent circuit based on the MBVD model, as shown in Figure 3. To characterize the loss from the electrodes, the MBVD model adds two additional loss resistors (i.e., *R*_0_ and *R_s_*) to obtain a more accurate model compared with the standard Butterworth–Van Dyke model. It consists of two network branches in parallel, where *R*_0_ represents the resistance associated with dielectric losses of the ME sensor, *R_s_* represents the resistance associated with electrical losses of electrode, *R_m_* denotes the resistance associated with mechanical losses, *L_m_* and *C_m_* denote the motional inductance and capacitance, *C*_0_ represents the static capacitance formed between top and bottom electrodes of the ME sensor.

The analytical expression of impedance Z(ω) and the electrical admittance Y(ω) for MBVD model are given by [27,28],

(7) $$Z\left(\omega \right)=R_{s}+\frac{(R_{0}+\frac{1}{j\omega C_{0}})\left(R_{m}+\frac{1}{j\omega C_{m}}+j\omega L_{m}\right)}{R_{0}+\frac{1}{j\omega C_{0}}+R_{m}+\frac{1}{j\omega C_{m}}+j\omega L_{m}}\;$$

(8) $$Y\left(\omega \right)=\frac{1}{\frac{1}{\frac{1}{R_{m}+\frac{1}{j\omega C_{m}}+j\omega L_{m}}+\frac{1}{\frac{1}{j\omega C_{0}}+R_{0}}}+R_{s}}\;$$

The series resonance frequency $f_{s}$ and antiresonance frequency $f_{a}$ can be expressed as [27,28],

(9) $$f_{s}=\frac{1}{2\pi \sqrt{L_{m}C_{m}}}$$

(10) $$\;\;\;f_{a}=\frac{1}{2\pi }\sqrt{\frac{C_{m}+C_{0}}{C_{0}L_{m}C_{m}}}=f_{s}\sqrt{\frac{C_{m}+C_{0}}{C_{0}}}$$

**Figure 4:**
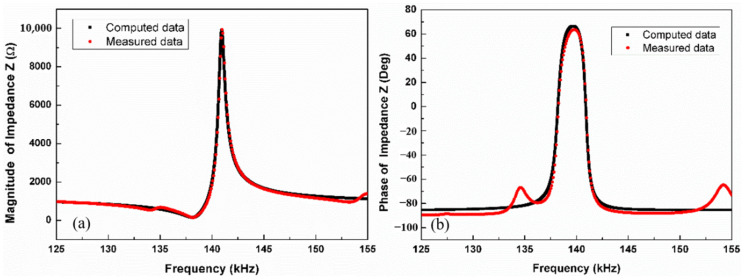
The (**a**) impedance and (**b**) phase angle of the ME sensor as a function of the electrical excitation frequencies ranged from 125 kHz to 155 kHz at *H*_dc_ = 30 Oe. The maximum RSDs of measured impedances and phase angles are 0.21% and 0.23%, respectively.

Using Equations (7), (9) and (10), the model parameter values of *C*_0_, *R*_0_, *R_s_*, *L_m_*, *C_m_* and *R_m_* are extracted from the measured Z. Table 1 lists all the extracted model parameters. To verify the MBVD model for further design of the conditioning circuit, the simulation of the model is implemented with the electrical simulator Agilent ADS. Figure 4 presents the computed impedance Z and phase based on the extracted model parameters at *H*_dc_ = 30 Oe, which shows a good agreement with the measured data.

**Figure 5:**
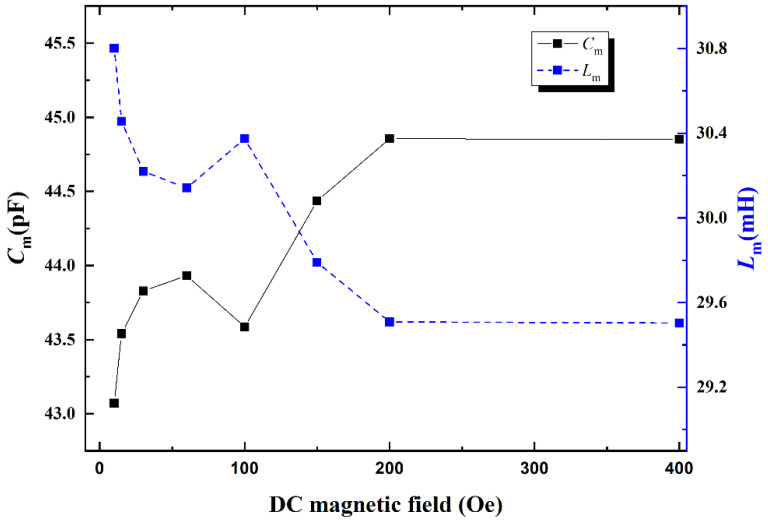
The *C_m_* and *L_m_* of the ME sensor as a function of DC magnetic field. Here, the maximum RSDs of *C_m_* and *L_m_* with multiple measurements are 0.23% and 0.25%, respectively.

**Figure 6:**
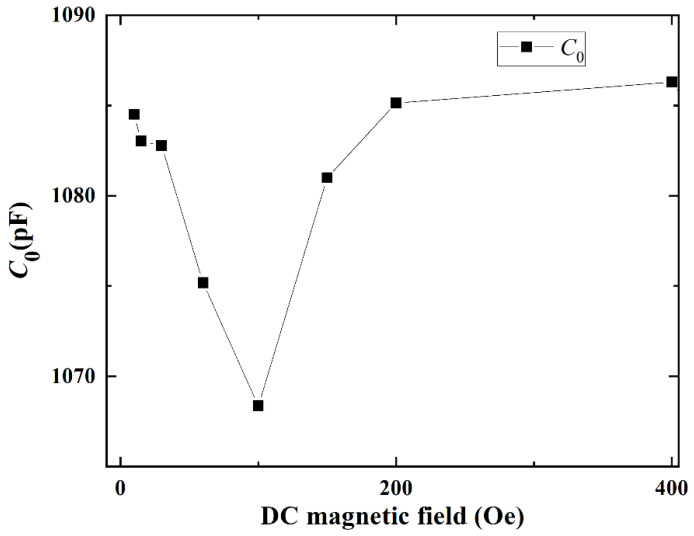
*C*_0_ of the ME sensor as function of DC magnetic field. The maximum RSD of *C*_0_ with multiple measurements is 0.25%.

**Figure 7:**
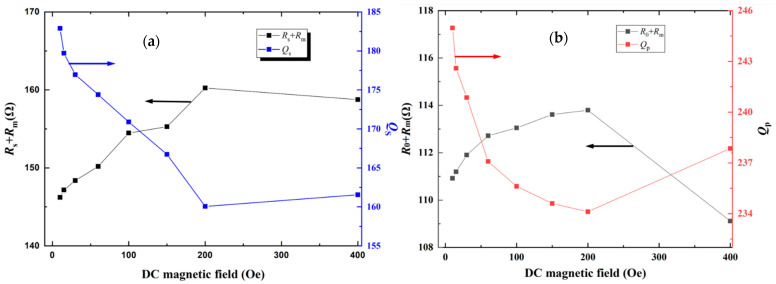
(**a**) The *Q*_s_ and *R_s_* + *R_m_* of the ME sensor as a function of DC magnetic field; (**b**) The *Q_p_* and *R*_0_ + *R_m_* of the ME sensor as a function of DC magnetic field. The maximum RSDs of *Q_s_* and *Q_p_* with multiple measurements are 0.22% and 0.21%, respectively. The maximum RSDs of *R_s_* + *R_m_* and *R*_0_ + *R_m_* with multiple measurements are 0.24% and 0.26%, respectively.

**Figure 8:**
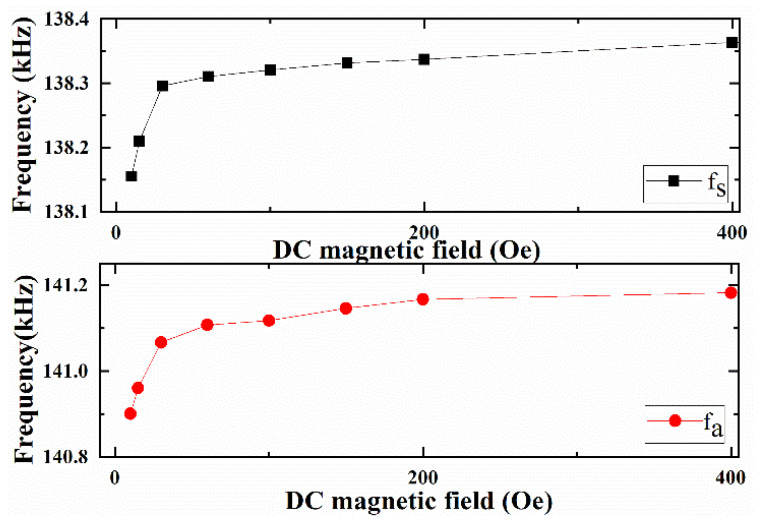
Resonance frequency *f_s_* and antiresonance frequency *f_a_* of ME resonator as a function of DC magnetic field. The maximum RSDs of *f_s_* and *f_a_* with multiple measurements are 0.23% and 0.27%, respectively.

According to the measured Z with DC bias magnetic field *H*_dc_, the corresponding equivalent circuit parameters (i.e., *C_m_*, *L_m_*, *C*_0,_ *Q_s_*, *R_s_* + *R_m_*, *f_s_* and *f_a_*) are calculated and analyzed as a function of *H*_dc_, as shown in Figure 5, Figure 6, Figure 7 and Figure 8, respectively.

Specifically, *C_m_* and *L_m_* are given by [27,28],

(11) $$L_{m}=\frac{1}{8}\frac{\rho Al_{t}}{l_{w}^{2}}{\left(\frac{s_{11}^{E}}{d_{31}}\right)}^{2}=\frac{1}{8}\frac{\rho ll_{t}}{l_{w}}{\left(\frac{s_{11}^{E}}{d_{31}}\right)}^{2}$$

(12) $$C_{m}=\frac{8}{\pi }\frac{A}{l_{t}}\frac{d_{31}^{2}}{s_{11}^{E}}=\frac{8}{\pi }\frac{ll_{w}}{l_{t}}\frac{d_{31}^{2}}{s_{11}^{E}}\;$$

where *d*_31_, $s_{11}^{E}$, ρ, *l_w_*, *l_t_* and *l* are the piezoelectric coefficient, elastic compliance coefficient, density, width, thickness and length of the ME sensor, respectively. $A=ll_{w}$ is the plate area.

It is obvious that the the length *l* and elastic compliance coefficient $s_{11}^{E}$ have strong influences on the *C_m_* and *L_m_* according to Equations (11) and (12). Specifically, due to the stress-strain coupling of interlayers, the magnetostrictive strain produced by FeSiB under varying *H*_dc_ results in the change in the length l and elastic compliance coefficient $s_{11}^{E}$ for piezoelectric material. As a result, the equivalent electrical parameters *C_m_* and *L_m_* of the ME sensor strongly depend on *H*_dc_ and vary in the opposite ways, as illustrated in Figure 5. This is due to the fact that *L_m_* and *C_m_* are proportional to ${\left(\frac{s_{11}^{E}}{d_{31}}\right)}^{2}$ and $\frac{d_{31}^{2}}{s_{11}^{E}}$, respectively.

Furthermore, *C_m_* is proportional to the length *l* of composite, whereas it is inverse proportional to the elastic compliance coefficient. Since the Young’s modulus is the inverse of elastic compliance coefficient, *C_m_* is determined by both the Young’s modulus *E* and length *l*. On one hand due to the stress-strain coupling of the interlayers, the length *l* increases quickly to a maximum value due to the large piezomagnetic coefficient *d*_33,*m*_ of FeSiB and then *l* reaches the saturation with further increasing *H*_dc_. On the other hand, the Young’s modulus *E* of the magnetostrictive layer and, corresponding, ME composite decrease initially to a minimum value with the increasing *H*_dc_, and then increases and reaches saturation at large *H*_dc_ when *H*_dc_ further increases [18]. When *H*_dc_ < 60 Oe, the magnetostriction does not attain to saturation, *C_m_* is affected by both Young’s modulus and the length *l*. However, the effect of length *l* on *C_m_* is more obvious than that of Young’s modulus due to the large *d*_33,*m*_ of FeSiB at the small *H*_dc_, which causes *C_m_* to increase with *H*_dc_ and reach a positive peak in low magnetic field *H*_dc_ = 60 Oe. When *H*_dc_ further increases above 60 Oe, the magnetostriction reaches saturation quickly; however, the Young’s modulus *E* still varies significantly and plays a dominant role in *C_m_*. Currently, Young’s modulus *E* and corresponding *C_m_* reach the local minimum values when *H*_dc_ further increases to 100 Oe, then *C_m_* gradually increases and reaches saturation with further increasing *H*_dc_ due to the variation in E, as shown in Figure 5.

Meanwhile the static capacitance *C*_0_ can be also expressed with the following expressions [28]:

(13) $$C_{0}=\frac{{A\varepsilon }_{r}\varepsilon_{0}}{l_{t}}$$

where $\varepsilon_{r}$ and $\varepsilon_{0}$ are the relative dielectric permittivity and vacuum permittivity of the piezoelectric material, respectively.

When *H*_dc_ is applied along the longitudinal direction of the ME sensor, the magnetostrictive material FeSiB expands with the increasing *H*_dc_, which changes dielectric permittivity of piezoelectric material due to the transferred magnetostrictive stress. Correspondingly, *C*_0_ varies with the DC magnetic field since *C*_0_ is strongly determined by the dielectric permittivity. Yao et al. [10] reported that the dielectric permittivity of Terfenol-D/PZT magnetoelectric composite at the resonant frequency decreased and then increased with increasing dc magnetic field. In this case, *C*_0_ varies in a similar trend as function of *H*_dc_ since *C*_0_ is proportional to the dielectric permittivity. Specifically, it is shown in Figure 6 that the static capacitance *C*_0_ of the ME sensor first decreases with the increasing *H*_dc_, and then gradually increases.

The *R_m_* is used to characterize the mechanical loss, which is subject to energy loss in the ME sensor. It can be given as [28],

(14) $$R_{m}=\frac{l_{t}^{3}}{8Ad_{31}^{2}}=\frac{l_{t}^{3}}{8ll_{w}d_{31}^{2}}$$

$R_{m}\;$ is primarily determined by the length *l*. Correspondingly, the variations in the length *l* due to the magnetostriction of FeSiB cause the variation in $R_{m}$ with *H*_dc_.

By analyzing the variation in equivalent circuit parameters (i.e., *C_m_*, *L_m_*, *C*_0,_ *R_m_* etc.) with *H*_dc_, it is found that the varying magnetostrictive strain of FeSiB with *H*_dc_ is the main reason for the *H*_dc_ dependences of equivalent circuit parameters. Furthermore, it is also noted that these equivalent circuit parameters depend on the actively vibrating area A, since *R_m_* and *L_m_* decrease with the enlarged area, whereas the capacitances *C*_0_ and *C_m_* increase with the enlarged area. Such relations are important for designing ME sensors.

Furthermore, the mechanical Quality factor (Q-factor) reflects the capability of the ME sensor to reserve mechanical energy and the corresponding loss of resonant circuit. *Q* is defined as the ratio of the stored energy to the dissipated energy per cycle during oscillation. According to Lakin’s method, the $Q_{s}$ at series resonance frequency $f_{s}$ and $Q_{p}$ at antiresonance frequency $f_{a}$ can be defined as [27,28],

(15) $$Q_{s}=\frac{2\pi f_{s}L_{m}}{R_{s}+R_{m}}\;\;$$

(16) $$Q_{p}=\frac{2\pi f_{p}L_{m}}{R_{0}+R_{m}}$$

On one hand, the smaller value of *R_S_* is desired to improve the effective mechanical Quality factor $Q_{s}$ of the ME sensor, according to Equation (15). Since *R_S_* represents the electrical loss of electrode, the type and quality of the electrode materials directly affect the $Q_{s}$ of the ME sensor. In this case, utilizing the electrode material with high acoustic impedance and low resistivity can reduce *R_S_* and improve the effective electromechanical coupling coefficient of the ME sensor. On the other hand, Equation (16) predicts that the high $Q_{p}$ value can be obtained when the ME sensor possesses a low $R_{0}$. Since the dielectric losses *R*_0_ of the ME sensor is mainly determined by the dielectric loss of piezoelectric material, it means the smaller dielectric loss results in the larger effective mechanical Quality factor $Q_{p}$ of the ME sensor. Additionally, from Equations (15) and (16), it is found that both $Q_{s}$ at resonance frequency and $Q_{p}$ at antiresonance frequency decrease with the increasing mechanical loss *R_m_*. Hence, the mechanical loss *R_m_* plays a primary role in the energy dissipations of the ME sensor.

Subsequently, the *Q_s_*, *Q_p_* and corresponding loss as a function of *H*_dc_ are experimentally investigated to verify and further understand the above theoretical analysis. It is known that *R_m_* depends on the mechanical energy dissipation $tan\delta_{mech}$ of the ME sensor [28], while *Q_s_* is inversely proportional to $tan\delta_{mech}$ [29]. When the DC magnetic field is applied, the mechanical energy dissipation *R_s_* + *R_m_* of magnetostrictive material FeSiB changes dramatically owing to the non-180° domain wall motions. This results in the varied mechanical quality factor *Q_s_* of the ME sensor with *H*_dc_, as shown in Figure 7a. Specifically, the quality factor (*Q_s_*) at the series resonance frequency $f_{s}$ decreases from 182 to the minimum value of 160 at the *H*_dc_ = 200 Oe and then gradually increases according to the MBVD model. Obviously, the variation in *Q_s_* is mainly attributed to the magnetic mechanical loss associated with magnetic domain wall movement and material damping of FeSiB.

Furthermore, the trends of $Q_{p}$ and *R*_0_ + *R_m_* as a function of *H*_dc_ are similar to that of $Q_{s}$ and *R_s_* + *R_m_*, as shown in Figure 7b. However, the magnitude of antiresonance mechanical quality factor $Q_{p}$ ranges from 234 to 245.6, which is higher than the resonance mechanical quality factor *Q_s_*. The differences between $Q_{p}$ and $Q_{s}$ were also reported by in previous literature [30,31]. Finally, the resonance frequency *f_s_* and antiresonance frequency *f_a_* of ME laminated sensor as a function of varied *H*_dc_ are investigated, as shown in Figure 8. Both *f*_s_ and *f*_a_ exhibit similar trends with *H*_dc_, which increases with the increasing DC bias field. The obvious shifts of resonance frequency *f_s_* and antiresonance frequency *f_a_* with *H*_dc_ indicate that *f_s_* and *f_a_* of the ME sensor are adjustable by varying the DC bias magnetic field.

## 4. Conclusions

In summary, the impedance of the ME sensor (i.e., FeSiB/PZT composite) as a function of DC bias magnetic field is experimentally measured and theoretically analyzed. Meanwhile, the simulation results with the MBVD model of the ME sensor agrees with the measured impedance Z accurately. Specifically, the dependences of extracted MBVD model parameters and the magnetoimpedance effects of the ME sensor on *H*_dc_ are observed, which result from the varied magnetostriction and the mechanical energy dissipation of magnetostrictive material FeSiB with *H*_dc_ due to the corresponding delta E effect and magnetostrictive effect. Furthermore, the influences of piezoelectric materials property and electrode on the MBVD model parameters are analyzed. The analysis of MBVD model for ME composite is beneficial to the design of analog front-end circuits for the corresponding magnetic sensor, which could further improve the LOD.

## Figures and Tables

**Table 1:** Parameters of the equivalent circuit model for ME resonator at *H*_dc_ of 30 Oe.

Model Parameters	*R_m_*	*L_m_*	*C_m_*	*R*_0_	*R_s_*	*C*_0_
values	88.3 Ω	30.2 mH	43.8 pF	20.8 Ω	60 Ω	1.08 nF

## Data Availability

Data sharing is not applicable to this article.
